# An investigation and analysis of the activities of daily living of older adults living at home in Ningxia Hui Autonomous Region of China: a cross-sectional study

**DOI:** 10.1186/s12877-020-01765-8

**Published:** 2020-09-25

**Authors:** Xiaofeng Xu, Lina Yang, Xiaohui Miao, Xiuying Hu

**Affiliations:** 1 The Trauma Center Ward 2 of West China Hospital/West China School of Nursing, Sichuan University, Chengdu, 610041 China; 2 Nursing department of the First People’s Hospital of Yinchuan, Yinchuan, China; 3 West China School of Nursing, Sichuan University, Chengdu, China; 4 Innovation Center of Nursing Research, West China School of Medicine/West China Hospital, Sichuan University, Chengdu, 610041 China

**Keywords:** Older adults, Living at home, Activities of daily living, Pension policy

## Abstract

**Background:**

To investigate the current situation regarding the activities of daily living (ADL) of older adults living at home in Ningxia Hui Autonomous Region of China and to analyze the associated factors of ADL performance so that we can provide references for the implementation of pension policies and long-term care insurance of older adults living at home.

**Methods:**

We surveyed a total of 1040 older adults who live at home and receive home-based care in Ningxia Hui Autonomous Region by a face-to-face evaluation. A logistic regression model was used to analyze the factors associated with ADL performance.

**Results:**

In the study, 82.79% of the older adults living at home can live independently. A total of 11.92% of the older adults have mild dysfunction, 4.33% have moderate dysfunction, and 0.96% have severe dysfunction. Multiple logistic analyses indicated that older adults with very difficult economic conditions (OR 3.212; 95% CI(1.209–8.534)) and without a spouse (OR 1.616; 95% CI(1.098–2.377)) were significantly associated with ADL limitations. In addition, the risks of ADL limitations in older adults aged 60–69 years and 70–79 years were 0.187 and 0.4307 times, respectively, that of older adults over 80 years old. The risk of ADL limitations in older adults of the Han nationality was 0.605 times that of the minority population. More highly educated and older adults without diseases have a lower risk of ADL limitations.

**Conclusions:**

Compared with the national average in China, the number of ADL limitations of older adults in Ningxia is greater and is associated with advanced age, ethnic minority status, low education level, low income, lacking a spouse and having diseases. As the number of older adults increases, maintaining and improving their ability to perform ADL and providing comfortable pension services and health services urgently need to be solved.

## Background

Since 1999, when societal aging began in China, population aging has been progressing [1]. The issues associated with population aging are also progressing. By the end of 2018, the number of older adults over the age of 60 in China reached 249.49 million, accounting for 17.9% of the total population [2]. The number of older adults is continuing to increase. The traditional and prevalent Chinese pension service is home-based care [3]. Home-based care is administered to older adults living in their own homes rather than in institutions [4]. The home-based care in China is similar to the community pension services in developed countries such as the United Kingdom. Community pension services take care of older adults in the community by professional staff or family, friends, neighbors, and community volunteers while using community resources [5]. However, compared with community pension services, home-based care in China often lacks mature community-supporting facilities, especially in remote areas with poor economic conditions and poor medical resources [3]. In areas with poor economic conditions and poor medical resources, such as the Ningxia Hui Autonomous Region of China, most of the young people work outside of the area. Their parents can only live alone or live with their spouses. Many older adults living in rural or remote areas do not have pension insurance [6]. Once they lose the activities of daily living (ADL), they will bring heavy care and economic burdens to their families. Therefore, the inability of older adults to perform ADL is particularly prominent. ADL comprise basic activities necessary for people to take care of themselves, maintain personal hygiene and carry out independent social activities in their daily lives, which are the most basic and common activities [7].

At the end of 2015, the number of disabled and semidisabled older adults in China was approximately 40.63 million, accounting for 18.3% of the older adults [8]. Long-term care for older adults who are disabled has become increasingly more important [9]. Facing the needs of a large number of disabled people for long-term care guarantees, the long-term care insurance system came into being in China. In 2016, 15 cities and two provinces (Shandong Province and Jilin Province) in China launched a pilot long-term care insurance system, focusing on solving the costs of basic living care and medical care for severely disabled people [10]. Ningxia Hui Autonomous Region currently has no pilot program for long-term care insurance. Therefore, disabled people, especially disabled older adults, still cannot enjoy long-term care insurance treatment.

According to the data, at the end of 2018, among the permanent residents of the Ningxia Hui Autonomous Region of China, there were 945,500 people aged 60 and over, accounting for 13.74% of the permanent resident population [11]. By the end of 2017, the size of the population aged 60 and over increased by 49,600, increasing the proportion of the older population by 0.6% [11]. As a minority autonomous region, Ningxia has unique characteristics. For example, ethnic minorities constitute 1/3 of the population of the region [12]. With the increasing number of older adults in Ningxia, the inability of older adults living at home to perform ADL during the day is also a prominent issue. With the continuous expansion of the scope of the pilot long-term care insurance, the pilot will be conducted in the Ningxia Hui Autonomous Region in the future. Then, investigating the ADL performance of older adults in the region and its associated factors can provide a reference for future long-term care insurance trials.

According to the World Health Organization (WHO), the ability to perform ADL is an essential indicator for the health status of older adults [13]. In addition, many studies have shown that older adults who lack ADL have a high psychological burden and a high risk for mortality [14]. Influenced by traditional Chinese concepts and cultural consciousness, home-based care has become the preferred way for older adults [15]. Therefore, when older adults cannot take care of themselves, there is a significant increase in the burden on families and caregivers [16]. Government and social support forces must intervene to care for older adults who cannot take care of themselves. However, before these interventions occur, it is necessary to investigate the ability of older adults to perform ADL in Ningxia. By analyzing the associated factors of ADL in older adults in Ningxia, we can provide a reference for the government to develop a better pension policy and to implement long-term care insurance. We can also provide some references to other developing countries and multiethnic countries like Ningxia Hui Autonomous Region of China on pensions.

## Methods

### Study design and survey tool

We consulted doctors, nurses, rehabilitators, dietitians, and other experts in fields related to aging before conducting the cross-sectional investigation. We decided to use the Barthel index (BI) as the assessment tool for this study. The BI is currently recognized worldwide as a tool for evaluating the ability of individuals to independently perform ADL. It was designed by Mahoney and Barthel in the mid-1950s [17]. The BI is a tool with excellent reliability and validity. The kappa values corresponding to the test-retest reliability and the split-half reliability are between − 1 and 1, and that of the split-half reliability is 0.903. The Cronbach’s alpha coefficient is 0.916. In addition, the Kaiser-Meyer-Olkin (KMO) statistic is 0.85 [18].

The BI consists of 10 items (feeding, bathing, grooming, dressing, bowel controlling, bladder controlling, toilet using, bed and chair transferring, mobility on level surfaces, stair climbing), and each item corresponds to three response options: full dependency, need help, and complete independence [19]. The total score can reach 100 points. The lower the score is, the higher the dependency of the individual. An overall score of 100 means that the respondent can take care of himself or herself and does not need help. An overall score of 60 to 99 indicates that the respondent has mild dysfunction and can complete some of the daily activities independently but that he or she needs some help. A total score of 41 to 59 points means that the respondent has moderate dysfunction and needs help completing daily activities. An overall score of fewer than 40 means that the respondent has severe dysfunction and that he or she cannot complete most ADL without help [20].

### Sampling

The required sample size was 700 to 1000 older adults according to the following formula: *n* = *z*^2^*p*(1-*p*)/{*e*^2^ + *z*^2^ *p*(1-*p*)/N}(*z* = 1.96; *P* represents the estimated value of the overall proportion; in this study, p represents the proportion of the older adults in the total population, i.e., *p* = 0.179; *e* is the allowable error limit and *e* = 0.03; N represents the number of older adults in China) [21]. This study is a cross-sectional survey. In addition, the sampling methods included stratified sampling, convenience sampling, and quota sampling. Stratified sampling involved categorizing each community health service center in a certain area as one of three types of communities: an urban community, an urban-rural integrated community, or a rural community. The convenience sampling method was used to extract communities and residents. In particular, 235 to 335 older adults were selected from each community for investigation. When the older adults were sampled with the convenience sampling method, 10% of the 235 to 335 older adults were required to be over 80 years old to achieve the purpose of quota sampling.

The inclusion criteria of the surveyed population included people who were 60 years old or over 60 years old and lived at home. The older adults included in the study could communicate without barriers after visual and auditory correction, provided informed consent and voluntarily participated in the survey. Older adults who had a mental illness, substance addiction, obvious memory or cognitive impairment, obvious hearing impairment, or language communication impairment and those who were unwilling to cooperate with the investigator were excluded.

### Data collection

The investigator conducted a face-to-face evaluation, and the respondent provided answers. The investigators were trained with the same protocol before the investigation, and the investigations were conducted at a time at which the respondents were not busy to achieve quality control. From September to December 2018, we surveyed a total of 1062 older adults, and 1040 valid questionnaires were returned, for a response rate of 97.9%.

### Statistical analysis

After the questionnaires were retrieved, we used an Excel table for preliminary data collation. Then, SPSS19.0 statistical software was used for the chi-squared test, rank-sum test, and logistic regression analysis. In the logistic regression analysis, missing values were 0. We assigned each independent variable (such as 60–69 years old =1, 70–79 years old = 2, ≥80 years old = 3) and added it to the logistic regression model and assigned dependent variables (live independently = 0, dysfunction = 1). *P* < 0.05 means the difference was statistically significant.

## Results

### Sociodemographic characteristics of the respondents and their ADL performance

The average age of the 1040 older adults living at home was 70.48 years, and there were 129 people over 80 years old. A total of 82.79% (861 older adults) of the older adults in the study living at home could live independently (older adults had no ADL limitations). A total of 11.92% (124 older adults) of the older adults had mild dysfunction, 4.33% (45 older adults) had moderate dysfunction, and 0.96% (10 older adults) had severe dysfunction. The older adults who were younger had better ADL abilities than those who were older. The older adults with Han nationality had better ADL abilities than those without Han nationality. The older adults who did not have religious beliefs (i.e., believed in a religion such as Christianity, Buddhism or Islam) had better ADL abilities than those who did have religious beliefs. The older adults with a high level of education had better ADL abilities than those with a low level of education. The older adults who had a spouse had better ADL abilities than those who did not have a spouse. The older adults with good economic status had better ADL abilities than those with poor economic status. In addition, the older adults with diseases had better ADL abilities than those without diseases. The difference was statistically significant (*P* < 0.05) (Table 1).

**Table 1:** The ADL performance in older adults living in Ningxia Hui Autonomous Region

		Number of people	Severe dysfunction N (%)	Moderate dysfunction N (%)	Mild dysfunction N (%)	Live independently N (%)	*χ*^*2*^_*/Z*_	*P*
Age^#^ (years)	60 ~ 69	505 (48.56%)	1 (0.20%)	13 (2.57%)	43 (8.51%)	448 (88.71%)	54.300	<0.001
	70 ~ 79	406 (39.04%)	6 (1.48%)	16 (3.94%)	49 (12.07%)	335 (82.51%)		
	≥80	129 (12.40%)	3 (2.33%)	16 (12.40%)	32 (24.81%)	78 (60.47%)		
Sex*	Male	527 (50.67%)	5 (0.95%)	18 (3.42%)	61 (11.57%)	443 (84.06%)	−1.169	0.242
	Female	513 (49.33%)	5 (0.97%)	27 (5.26%)	63 (12.28%)	418 (81.48%)		
Ethnicity* ^①^	Han nationality	854 (82.12%)	6 (0.70%)	25 (2.93%)	98 (11.48%)	725 (84.89%)	−4.152	<0.001
	minority	186 (17.88%)	4 (2.15%)	20 (10.75%)	26 (13.98%)	136 (73.12%)		
Religious beliefs * ^②^	none	542 (52.12%)	4 (0.74%)	16 (2.95%)	61 (11.25%)	461 (85.06%)	−2.146	0.032
	have	498 (47.88%)	6 (1.20%)	29 (5.82%)	63 (12.65%)	400 (80.32%)		
Educational level^#^	illiteracy	299 (28.75%)	6 (2.01%)	18 (6.02%)	32 (10.70%)	243 (81.27%)	31.895	0.007
	primary school	303 (29.13%)	2 (0.66%)	12 (3.96%)	36 (11.88%)	253 (83.50%)		
	junior high school	189 (18.17%)	1 (0.53%)	5 (2.65%)	19 (10.05%)	164 (86.77%)		
	Secondary or high school	108 (10.38%)	1 (0.93%)	3 (2.78%)	13 (12.04%)	91 (84.26%)		
	College or undergraduate	124 (11.92%)	0 (0.00%)	3 (2.42%)	20 (16.13%)	101 (81.45%)		
	Postgraduate or above	17 (1.63%)	0 (0.00%)	4 (23.53%)	4 (23.53%)	9 (52.94%)		
With a spouse* ^③^	no	237 (22.79%)	2 (0.84%)	20 (8.44%)	39 (16.46%)	176 (74.26%)	−4.029	<0.001
	yes	803 (77.21%)	8 (1.00%)	25 (3.11%)	85 (10.59%)	685 (85.31%)		
Way of living*^④^	Living alone	48 (4.62%)	2 (4.17%)	3 (6.25%)	4 (8.33%)	39 (81.25%)	−0.421	0.674
	Living with family	755 (72.60%)	6 (0.79%)	22 (2.91%)	81 (10.73%)	646 (85.56%)		
Economic situation ^# ⑤^	Very difficult	42 (4.04%)	1 (2.38%)	5 (11.90%)	8 (19.05%)	28 (66.67%)	43.958	<0.001
	Slightly difficult	274 (26.35%)	5 (1.82%)	21 (7.66%)	43 (15.69%)	205 (74.82%)		
	Moderate	443 (42.60%)	4 (0.90%)	7 (1.58%)	48 (10.84%)	384 (86.68%)		
	Slightly favorable	201 (19.33%)	0(.00%)	5 (2.49%)	18 (8.96%)	178 (88.56%)		
	Favorable	80 (7.69%)	0 (0.00%)	7 (8.75%)	7 (8.75%)	66 (82.50%)		
Medical insurance* ^⑥^	none	71 (6.83%)	0 (0.00%)	3 (4.23%)	5 (7.04%)	63 (88.73%)	−1.348	0.178
	have	969 (93.17%)	10 (1.03%)	42 (4.33%)	119 (12.28%)	798 (82.35%)		
Body Mass Index^#^ (BMI) ^⑦^	<18.5	31 (2.98%)	0 (0.00%)	4 (12.90%)	5 (16.13%)	22 (70.97%)	10.810	0.289
	18.5–23.9	603 (57.98%)	7 (1.16%)	20 (3.32%)	70 (11.61%)	506 (83.91%)		
	24–27.9	338 (32.50%)	3 (0.89%)	19 (5.62%)	40 (11.83%)	276 (81.66%)		
	≥28	68 (6.54%)	0 (0.00%)	2 (2.94%)	9 (13.24%)	57 (83.82%)		
Diseases * ^⑧^	none	104 (10.00%)	2 (1.92%)	8 (7.69%)	22 (21.25%)	72 (69.23%)	−3.855	<0.001
	have	936 (90.00%)	8 (0.85%)	37 (3.95%)	102 (10.90%)	789 (84.29%)		

Note: * indicates the use of the rank-sum test; # indicates the use of the chi-squared test

①the fact or state of belonging to a social group that has a common national or cultural tradition;②the person believes in a religion such as Christianity, Buddhism or Islam; ③does the older adult currently have a spouse; ④whether the older adult lives with others; ⑤balance of family members’ income and expenditure; ⑥Does the older adult have medical insurance; ⑦a weight-to-height ratio, calculated by dividing one’s weight in kilograms by the square of one’s height in meters and used as an indicator of obesity and underweight; ⑧whether the older adults have a disease

### The factors associated with ADL performance in older adults in the Ningxia region

Factors that may be associated with older adults’ ability to perform ADL in Ningxia, such as a person’s age, ethnicity, religious beliefs, educational level, marriage status, and economic situation and whether he or she has a disease, were treated as independent variables. We conducted multivariate logistic binary regression analysis with ability to perform ADL as the dependent variable. The results showed that the factors associating with ADL performance in older adults living in Ningxia included one’s age, ethnicity, educational level, economic situation, marriage status, and whether the older adult had a disease (Table 2). The risks of ADL limitations in older adults aged 60–69 years and 70–79 years were 0.187 and 0.4307 times that of older adults over 80 years old. The risk of ADL limitations in the older adults of the Han nationality was 0.605 times that of the minority population. The risks of ADL limitations in the older adults who completed primary school, junior high school, secondary school or high school, junior college or undergraduate education, and postgraduate education or above were 0.142, 0.128, 0.122, 0.158, and 0.241 times that of the illiterate older adults, respectively. The risk of ADL limitations in older adults with very difficult and slightly difficult economic conditions were 3.212 and 2.191 times that of the economically wealthy older adults, respectively. The risk of ADL limitations in older adults without a spouse was 1.616 times that of older adults with a spouse. The risk of ADL limitations in older adults without a disease living at home was 0.409 times that of older adults with diseases.

**Table 2:** Multiple logistic regression analysis of factors associated with ADL limitations in older adults in the Ningxia Hui Autonomous Region

Variable	*b*	*Standard error*	*WalsX*^*2*^	*P*	*OR*	*95% CI*
Age (years)						
≥ 80					1 (reference)	
60 ~ 69	−1.674	0.245	46.753	<0.001	0.187	(0.116–0.303)
70 ~ 79	− 1.180	0.238	24.695	<0.001	0.307	(0.193–0.489)
ethnic						
minority					1(reference)	
Han nationality	−0.503	0.228	4.858	0.028	0.605	(0.386–0.946)
Religious beliefs						
have					1 (reference)	
none	−0.134	0.201	0.445	0.505	0.874	(0.589–1.297)
Educational level						
Illiteracy					1 (reference)	
Primary school	−0.945	0.588	11.046	0.001	0.142	(0.045–0.449)
Junior high school	−2.052	0.593	11.959	0.001	0.128	(0.040–0.411)
Secondary or high school	−2.105	0.613	11.790	0.001	0.122	(0.037–0.405)
College or undergraduate	−1.846	0.631	8.546	0.003	0.158	(0.046–0.544)
Postgraduate or above	−1.422	0.599	5.628	0.018	0.241	(0.074–0.781)
Economic situation						
Abundant					1 (reference)	
Very difficult	1.167	0.499	5.476	0.019	3.212	(1.209–8.534)
Slightly difficult	0.784	0.388	4.086	0.043	2.191	(1.024–4.688)
Moderate	−0.021	0.380	0.003	0.955	0.979	(0.465–2.060)
Slightly favorable	−0.281	0.410	0.470	0.493	0.755	(0.338–1.686)
With a spouse						
yes					1 (reference)	
no	0.480	0.197	5.926	0.015	1.616	(1.098–2.377)
Diseases						
have					1 (reference)	
None	−0.894	0.251	12.683	<0.001	0.409	(0.250–0.669)

## Discussion

This study shows that there are 861 people (82.79%) living at home who can live independently in Ningxia, China. In addition, there are 179 people (17.21%) who are completely or partially unable to live independently. According to a survey on ADL abilities in older adults living in other regions of China, 8% of the older adults living in Guangdong, Shanghai, Shanxi, Hubei, and Heilongjiang provinces cannot live independently [22]. A study investigating the ADL abilities of older adults living in Beijing, China found that 87.9% of them can live independently [23]. According to a survey conducted by the National Committee on aging, the number of older adults who live independently in urban areas in China is 85.40%, and in rural areas, it is 79.00%. Therefore, compared with the national average or that of other regions in China, the number of ADL limitations of older adults in Ningxia is greater [24]. At present, the aging population in Ningxia is increasing. At the end of 2018, the number of individuals aged 60 and over was 945,500, accounting for 13.74% of the total population [11]. With the increasing number of older adults and the impact of diseases, the number of disabled older adults will also increase, and the number of ADL limitations will continue to increase. This finding reminds us to address the level of ability to perform ADL by older adults, especially older adults living at home, and to address the daily needs of the disabled older adults. With the gradual increase of disabled older adults in Ningxia, the implementation of related disability insurance policies, such as long-term care insurance, can provide assistance to a large number of disabled older adults. The government should formulate assessment and judgment criteria for measuring the incapacity of older adults in Ningxia Hui Autonomous Region as soon as possible and formulate corresponding subsidy policies. Such policies could alleviate the huge economic and care burden brought by disabled older adults in Ningxia.

The factors associating with ADL performance in the older adults are complex. The individual’s natural attributes, living environment, economic status, disease status, and injury status are the main factors affecting ADL performance in older adults [25]. The results of this study show that one’s age, ethnicity, educational level, economic situation, marriage status, and whether one has a disease are all factors associating with their ability to perform ADL independently.

Several studies have shown that older adults’ ability to perform ADL is negatively correlated with age. The older the individual is, the lower the ability to perform ADL. In addition, the rate of decline in the ability to perform ADL increases with age [26, 27]. The survey also found that the proportion of individuals over 80 years old with ADL difficulty was significantly higher than that of individuals who were 60–79 years old, and the difference was statistically significant (*P* < 0.001). With an increase in age, the functions of the older adults gradually decline, and problems associated with comorbidities also appear. The ability to perform ADL also decreases rapidly, and the proportion of individuals with disabilities increases. Therefore, age is associated with older adults’ ability to perform ADLs. The pension policy, such as a long-term care insurance system, should focus on the disability of older adults, especially disability assessments and subsidies for older adults over 80 years old. For older adults over 80 years old, community workers should also conduct disability assessments and functional maintenance exercises on time. Once an individual becomes incapacitated, life care and financial subsidies should be carried out in time.

Ningxia Hui Autonomous Region is a place where many ethnic groups live together, including Han, Hui, Uygur, Dongxiang, Kazak, Salar, and Baoan individuals. This study found that ethnicity is associated with ADL performance in older adults. The ADL performance of members of the Han nationality is better than that of ethnic minorities, and the risk of ADL limitations in members of the Han nationality is much lower than that of ethnic minorities. This result is related to the living environment of ethnic minorities in Ningxia. Many ethnic minority older adults live in relatively remote or rural areas, and they do not have pension insurance [28]. They are unable to obtain good health care and endowment insurance. Insurance similar to the long-term care insurance system can provide basic protection for ethnic minority older adults when they are disabled. For older ethnic minorities, regular ADL performance assessments should become one of the works of community workers to provide them with timely help.

In addition, the level of education and economic status was also associated with the ADL performance of older adults living at home. The higher the level of education is, the better the ADL performance of the older adults. According to the results of this study, older adults who have received an education have a lower risk of ADL limitations than those who are illiterate. The risk of ADL disability is increasing among older adults with a low socioeconomic status. An older adult follow-up survey in Taiwan, China showed that there was a negative correlation between social class and ADL performance after controlling for the level of education [29]. The results of this study also show that the ADL performance of older adults with good economic conditions is significantly higher than that of older adults with poor economic conditions. It may be that well-educated and high-income older adults have a higher capacity and more resources to deal with a disability. In addition, they are less likely to be severely disabled than those with a low level of education and low income. Older adults with poor economic status are often lacking support from a social health care system or can obtain only limited medical resources. Therefore, for older adults with low income and education levels, the long-term care insurance system should provide more subsidies. After the disability assessment of older adults living at home, financial subsidies are provided according to the disability level and economic status of older adults.

The study also found that older adults with spouses have better ADL performance than those without spouses. Most older adults with spouses can support each other and help each other to achieve self-care. In this study, there were 789 older adults (84.29%) who had a disease and could live independently, and only 72 people (69.23%) were in good physical health and could live independently. Studies have found that chronic diseases, such as diabetes, cataracts, strokes, and bronchial diseases, are the leading causes of the decline in ADL in the older adults in China. Alzheimer’s disease is also one of the severe diseases affecting the ADL performance of older adults [30]. The results of this study are inconsistent with the results of previous studies. It may be that more older adults with diseases than older adults without diseases participated in the survey. Therefore, we conducted a multivariate analysis. The results showed that the risk of ADL limitations in older adults with a disease was higher than that of those without the disease. This result is consistent with previous research conclusions. A variety of diseases leading to declines in sensory function, including vision, hearing, swallowing ability, or physical disabilities, in older adults can cause a severe decline in ADL performance, hindering older adults living at home from living independently. It is recommended that Ningxia accelerate the establishment of a long-term pension insurance system to include family members in paying caregivers to reduce the pension burden of the whole society [31].

### Limitations

This study does have some limitations. Due to resource and time constraints, this study did not investigate the current status of ADL performance in older adults in all cities in Ningxia. Only three cities were selected for investigation. A more comprehensive survey will be conducted in future research to complement the findings of this study.

## Conclusion

In this survey, compared with the national average in China, the number of ADL limitations of older adults in Ningxia is greater. Moreover, the factors associated with ADL performance are complex. Advanced age, ethnic minority status, low educational level, low income, lacking a spouse and having diseases are all associated with ADL limitations. With the acceleration of the aging process and the increasing number of older adults in Ningxia, how to maintain and improve ADL performance among older adults, especially older adults living at home, and how to provide comfortable pension services and health services urgently need to be solved.

## Data Availability

The datasets used or analyzed during the current study are available from the corresponding author upon reasonable request.

## Abbreviations

ADL: Activities of daily living
WHO: World health organization
BI: Barthel index
KMO: Kaiser-meyer-olkin
